# Supplementary material for: 2FAST2Q: a general-purpose sequence search and counting program for FASTQ files
Source: PeerJ. 2022 Oct 25;10:e14041. doi: 10.7717/peerj.14041 (PMC9615965; doi:10.7717/peerj.14041)
Supplement: Figure S1 [file peerj-10-14041-s001.pdf]

D:\UNIL\Python_Programs\2FAST2Q\dist\2FAST2Q_windows.exe

```
Version: 2.3.4

Running in align and count mode with the following parameters:
0 mismatch allowed
Minimal Phred Score per bp >= 30
Feature length: 20
Read alignment start position: 0

All data will be saved into C:/Users/afons/Downloads/bikard_fastq/2FAST2Q_output_2021_11_24_10_49_13\2FAST2Q_output_2021_12_20_13_17_45

Loading Features

11629 different features were provided.

Processing 118 files. Please hold.

Processing file 4 out of 118:  63%|###############7         | 90870601/143945160 [00:05<00:03, 16673030.70characters/s]
Processing file 12 out of 118:   5%|#1                      | 14787364/298122739 [00:00<00:17, 16509475.69characters/s]
Processing file 6 out of 118:  50%|############3            | 87087627/175743839 [00:05<00:05, 16110963.00characters/s]
Processing file 11 out of 118:   9%|##2                     | 26539623/284003755 [00:01<00:15, 16497925.05characters/s]
Processing file 3 out of 118:  79%|###################7     | 89092403/112897070 [00:05<00:01, 16359410.16characters/s]
Processing file 5 out of 118:  58%|##############5          | 90612488/155978997 [00:05<00:03, 16974129.56characters/s]
Processing file 9 out of 118:  37%|#########2               | 86665482/234426059 [00:05<00:08, 16576674.86characters/s]
Processing file 7 out of 118:  47%|###########6             | 88797884/189768453 [00:05<00:06, 16529244.99characters/s]
Processing file 10 out of 118:  35%|########3               | 90829008/262005192 [00:05<00:09, 17453406.44characters/s]
Processing file 8 out of 118:  44%|###########              | 86629758/195837051 [00:05<00:06, 16187016.99characters/s]
```
