# Supplementary material for: 2FAST2Q: a general-purpose sequence search and counting program for FASTQ files
Source: PeerJ. 2022 Oct 25;10:e14041. doi: 10.7717/peerj.14041 (PMC9615965; doi:10.7717/peerj.14041)
Supplement: Figure S2 — When dealing with mismatches, 2FAST2Q will preemptively convert all the input feature sequences into their respective binary integer format using 8bits encoding. This step ensues faster downstream processing, decreases RAM usage, and allows mismatches to be calculated by a simple subtraction performed at machine code speed. [file peerj-10-14041-s002.pdf]

Conversion from string to corresponding integer 8 format	A	T	G
	65	84	71
	A	T	C
	65	84	67
position wise subtraction	65-65	84-84	71-67
The non 0 result position is the mismatch location	0	0	4
