# Supplementary figures and images for: 2FAST2Q: a general-purpose sequence search and counting program for FASTQ files
Source: PeerJ. 2022 Oct 25;10:e14041. doi: 10.7717/peerj.14041 (PMC9615965; doi:10.7717/peerj.14041)

Read abundance correlation between sgRNAs

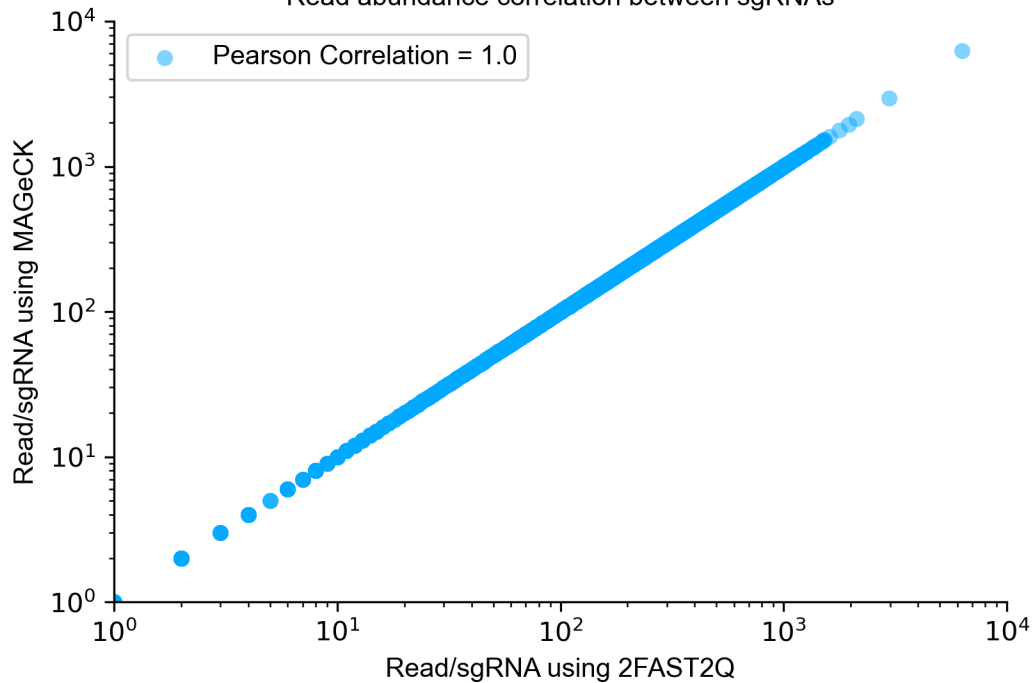

Supplement: Figure S3 — The total read counts were obtained using MAGeCK (y axis) and 2FAST2Q (x axis). Pearson correlation is also shown. [file peerj-10-14041-s003.pdf]
